# Supplementary material for: scVGAMF: a novel imputation method for scRNA-seq data by integrating linear and non-linear features
Source: Brief Bioinform. 2025 Oct 27;26(5):bbaf562. doi: 10.1093/bib/bbaf562 (PMC12554638; doi:10.1093/bib/bbaf562)
Supplement: supplementary_file_BIB-25_1447_bbaf562 [file supplementary_file_bib-25_1447_bbaf562.pdf]

Supplementary materials

1 Update rules of the model

To optimize the objective, we adopt the alternating-direction method of multipliers (ADMM) algorithm [1], updating W and H in an alternating way. First, H is kept fixed, and the subproblem with respect to W is:

$$\mathcal{L}(W) = \frac{1}{2}\sum_{i,j}\left(X_{ij} - \sum_{k} W_{ik}H_{kj}\right)^2 + \frac{\omega_1}{2}\sum_{i,k} W_{ik}^2 \tag{1}$$

According to the Majorization-Minimization (MM) and Bregman divergence [2], we construct auxiliary functions $G(W_{ik}|W'_{ik})$ for each element W_{ik}:

$$G(W_{ik}|W'_{ik}) = \mathcal{L}(W'_{ik}) + \sum_{i,j} \frac{(W_{ik}H_{kj} - W'_{ik}H_{kj})^2}{2\sum_{k'} W'_{ik'}H_{k'j}} + \frac{\omega_1}{2}W_{ik}^2 \tag{2}$$

The derivation of $G(W_{ik}|W'_{ik})$ with respect to W_{ik} is:

$$\frac{\partial G}{\partial W_{ik}} = \sum_{j} \frac{H_{kj}\left(\sum_{k'} W_{ik'}H_{k'j} - X_{ij}\right)}{\sum_{k'} W'_{ik'}H_{k'j}} + \omega_1 W_{ik} \tag{3}$$

Setting the derivative to zero, the result is as follows.

$$W_{ik} = W'_{ik} \cdot \frac{\sum_j X_{ij}H_{kj} / \sum_{k'} W'_{ik'}H_{k'j}}{\sum_j H_{kj}^2 + \omega_1} \tag{4}$$

Similarly, we update W and H using the multiplicative update rule:

$$W \leftarrow W \cdot \frac{XH^T}{WHH^T + \omega_1 W} \tag{5}$$

$$H \leftarrow H \cdot \frac{W^T V}{W^T W H + \omega_2 H} \tag{6}$$

2 Baseline methods

Below is a brief overview of these methods:

- Single-cell Imputation (**scImpute** [3]): It applies a gamma-Gaussian mixture model to learn gene expression pattern across similar cells, and uses non-negative least squares regression to impute potential missing values.
- Single-cell Analysis Via Expression Recovery (**SAVER** [4]): SAVER tackles the challenges of data sparsity and high noise levels by using a Poisson-Gamma distribution model and applies Poisson-lasso regression to estimate missing values.
- Two-step Imputation (**TsImpute** [5]): TsImpute adopts a zero-inflated negative binomial distribution to distinguish dropouts from true zeros and imputes missing values based on weighted distances in the final iteration.
- Markov Affinity-based Graph Imputation of Cells (**MAGIC** [6]): MAGIC is a data smoothing method and applies Markov random walk on a cell-cell similarity graph to estimate missing values.
- Imputing Dropout Events in Single-Cell RNA-Sequencing Data (**DrImpute** [7]): Identifies similar cells through clustering and imputes missing data by averaging gene expression values across these cells.
- Single-cell Reconstruction via Matrix Decomposition (**scRMD** [8]): scRMD employs robust matrix decomposition as its objective function, enabling the reconstruction of missing values and noise filtering.
- Single-cell Low-rank Tensor Completion (**scLRTC** [9]): scLRTC models gene expression data as a multi-dimensional tensor, assuming a low-rank structure within the tensor that reflects biological correlations.
- Adaptive Local Relevance Analysis (**ALRA** [10]): ALRA utilizes singular value decomposition to obtain a low-rank approximation of the gene expression matrix, thereby capturing missing values of expressed genes.
- Contrastive Learning-based Imputation (**CL-Impute** [11]): CL-Impute leverages contrastive learning to learn cell representations from the self-perspective of dropout events, with a self-attention network captures cell relationships from a global perspective.
- Single-cell Variational Graph Autoencoder (**scVGAE** [12]): scVGAE integrates ZINBLoss and GCN to improve imputation performance while preserving cellular information integrity.

Table S1 provides a summary of these methods, including their publication year, model type, software, and availability.

Table S1: Summary of existing imputation methods for scRNA-seq data.

Method	Year	Type	Software	Availability
**scImpute**	2018	Statistical model	R	https://github.com/Vivianstats/scImpute
**SAVER**	2018	Statistical model	R	https://github.com/mohuangx/SAVER
**TsImpute**	2023	Statistical model	R	https://github.com/ZhengWeihuaYNU/tsImpute
**MAGIC**	2018	Data smoothing	R/Python	https://github.com/KrishnaswamyLab/MAGIC
**DrImpute**	2018	Data smoothing	R	https://github.com/gongx030/DrImpute
**scRMD**	2020	Matrix decomposition	R	https://github.com/XiDsLab/scRMD
**ScLRTC**	2021	Matrix decomposition	MATLAB	https://github.com/jianghuaijie/scLRTC
**ALRA**	2022	Matrix decomposition	R	https://github.com/KlugerLab/ALRA
**CL-Impute**	2023	Deep Learning	Python	https://github.com/yuchen21-web/Imputation-for-scRNA-seq
**scVGAE**	2024	Deep Learning	Python	https://github.com/inoue0426/scVGAE

3 Functional enrichment analysis

In studies of human embryonic stem cell (ES cell) differentiation into definitive endoderm cells (DE cells), scRNA-seq captures cellular heterogeneity. However, dropout events can obscure critical gene expression signals and introduce bias in functional enrichment analysis. To evaluate the preservation of biological information by scVGAMF in scRNA-seq data, Gene Ontology (GO) enrichment analysis was performed on the top 300 differentially expressed genes (DEGs) identified from both the raw and scVGAMF-imputed datasets. The GO enrichment results were generated using the clusterProfiler R package [13]. Figure S1 lists the top 10 GO terms after imputation, all of which are highly relevant to DE differentiation. Examples include "endoderm formation" (GO:0001706), "endoderm development" (GO:0007492) and "endodermal cell differentiation" (GO:0035987). These results demonstrate that scVGAMF effectively imputes missing values while preserving biologically relevant information, which is consistent with the known biology of the system.

The differentiation of ES cells into DE cells is a critical event in early embryonic development. This process proceeds sequentially through stages of cell fate determination, axis polarity establishment, and endoderm specification [14–16]. Three novel biological process (BP) terms emerged after imputation: forebrain development (GO:0030900), axis specification (GO:0009798), and endodermal cell differentiation (GO:0035987). These terms correspond to events involved in refined cell lineage specification. For research focused on the differentiation of human ES cells into DE cells, endodermal cell differentiation (GO:0035987) represents a core biological process. The emergence of this term following imputation demonstrates the method's capacity to restore key signals regulating cell fate determination. Furthermore, the detection of forebrain development (GO:0030900) and axis specification (GO:0009798) suggests that imputation unmasks region-specific signals previously buried under technical noise. In contrast, biological processes identified solely in the raw data—namely heart morphogenesis (GO:0003007), stem cell differentiation (GO:0048863), and pattern specification process (GO:0007389)—are broader in scope. Stem cell differentiation (GO:0048863) is a fundamental attribute of ES cells but lacks specificity to the DE differentiation stage. Heart morphogenesis (GO:0003007) shows limited relevance to core trajectories of endoderm differentiation, which give rise to precursors of the digestive and respiratory system. Pattern specification process (GO:0007389) is a general mechanism of embryonic development and therefore provides limited cell type-specific information.

The differentiation of ES cells into DE cells is a highly regulated directional process. The

novel biological terms identified following imputation are directly associated with germ layer specification (particularly endoderm) and embryonic axis establishment—processes critical for DE differentiation. In contrast, terms identified from raw data show only weak associations with the target differentiation pathway (heart development). These findings demonstrate that incomplete gene expression signals in raw data can lead to biased functional annotations.

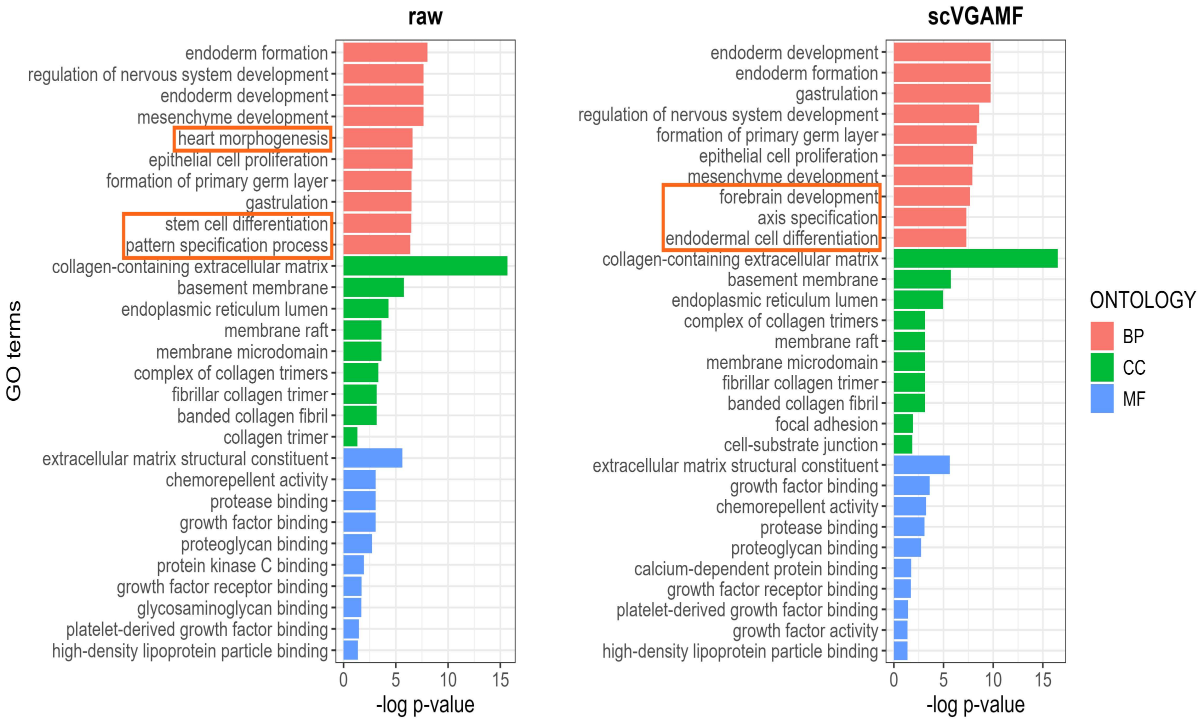

Fig. S1: The top 10 enriched BP, CC, and MF categories sorted by adjusted P-values on raw data and scVGAMF-imputed data.

4 Alternative methodologies

4.1 Cell clustering using K-Means

For cluster analysis, we employed K-Means [17] clustering as an alternative approach. The standardized matrix generated by the scVGAE method was incompatible with K-Means clustering and was therefore excluded from comparative analysis. Each method was executed independently over ten times, and the mean ARI and NMI values are reported in Figure S2. scVGAMF achieved the highest ARI and NMI scores on the Romanov dataset, ranked second on both the Baron_human and Baron_mouse datasets, and third on the Zeisel dataset. Notably, DrImpute outperformed SC3 when coupled with K-Means clustering. This may be due to DrImpute's iterative use of K-Means to identify cell subgroups, followed by cluster-based imputation using mean expression values.

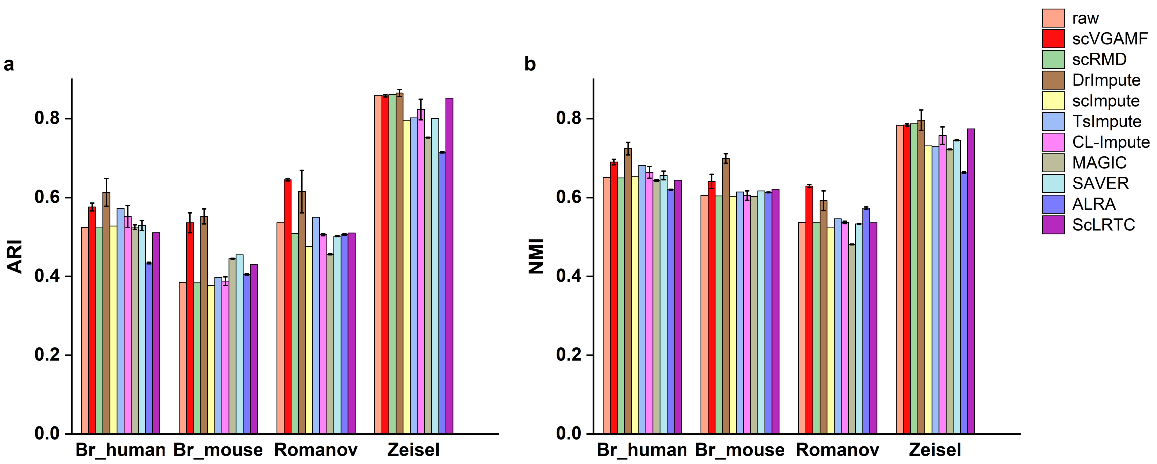

Fig. S2: Clustering performance across four real-world datasets. (a) ARI and (b) NMI scores for different clustering methods. Higher values indicate better agreement with ground-truth labels. Error bars represent standard deviation over ten runs.

4.2 Pseudotime inference using TSCAN

For pseudotime trajectory inference, we employed TSCAN [18] as an alternative method. Each method was independently run ten times, and the mean POS and Kendall's rank correlation coefficients were computed. Since TSCAN algorithm includes a highly variable gene (HVG) selection step, certain imputation methods fail to preserve differential expression patterns, making pseudotime trajectory inference impossible. Furthermore, when imputation methods produce inconsistent results across replicates, variations in HVG selection may cause significant fluctuations in the final trajectory inference results. As shown in Figure S3, scVGAMF achieved the highest POS (0.934) on the Time course dataset and ranked thrid in Kendall rank correlation scores (0.734), slightly inferior to scImpute and scLRTC. On the Petropoulos dataset (Figure S4), scVGAMF yielded median performance in both POS (0.874) and Kendall's correlation (0.712), yet significantly exceeded the raw data (POS: 0.774; correlation: 0.559), confirming its utility in enhancing trajectory inference.

4.3 Differential expression analysis using DESeq2

In terms of differential gene expression analysis, we employed DESeq2 [19] as an alternative analytical method. Figure S5(a) shows the Spearman correlation coefficients between adjusted P-values from bulk data and imputed(raw) data. The results show that scVGAMF identified differentially expressed (DE) genes with the second-highest consistency compared to the 1,160 DE genes identified from bulk data. In contrast, methods such as CL-Impute and ALRA failed to improve the spearman correlation compared to the raw data. We selected the top 300 DE genes based on adjusted P-values from the bulk data as the gold standard reference. These reference genes were then compared with genes identified from both raw and imputed data to evaluate the performance of different imputation methods. Figure S5(b) illustrates the overlap between reference DE genes from bulk data and those detected in raw versus imputed data. MAGIC, scImpute, and scVGAMF were among the top three methods, as they identify the largest number of gold standard DE genes. Additionally,

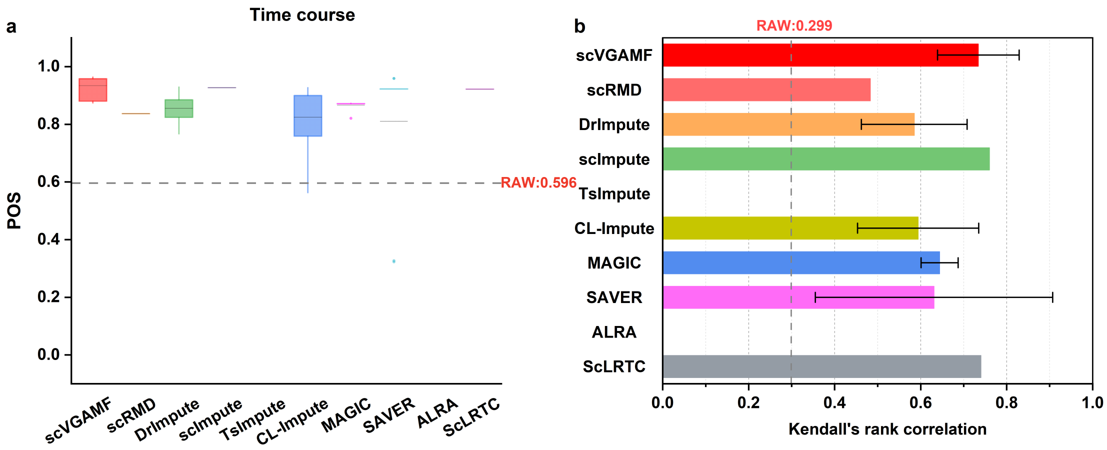

Fig. S3: Evaluation of trajectory inference on the Time course dataset. (a) POS scores. (b) Kendall's rank correlation.

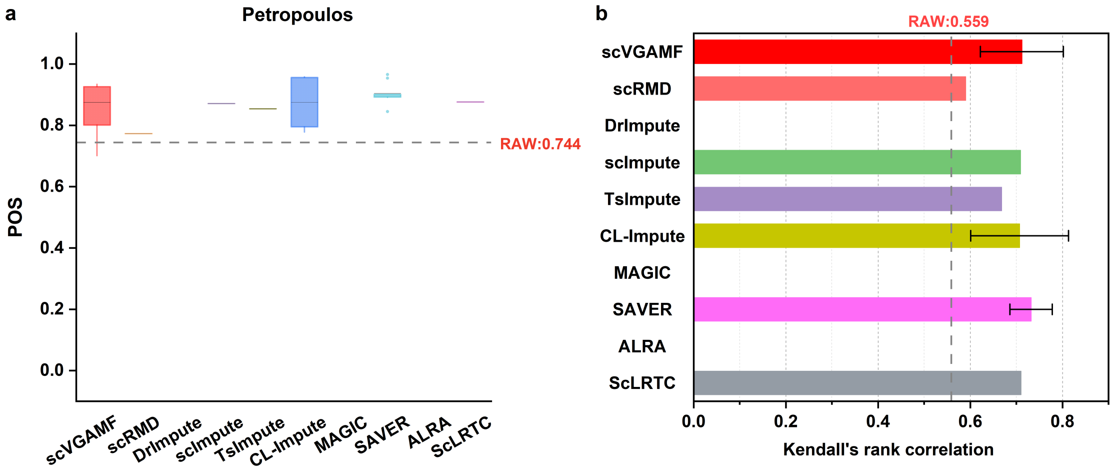

Fig. S4: Evaluation of trajectory inference on the Petropoulos dataset. (a) POS scores. (b) Kendall rank correlation.

we visualized both raw and imputed data using volcano plots (Figure S5(c)). DE cells are enriched in genes such as LHX1, NODAL, ERBB4, LEFTY1 and PAX6. scVGAMF showed higher -log(P) values for these genes compared to the raw data.

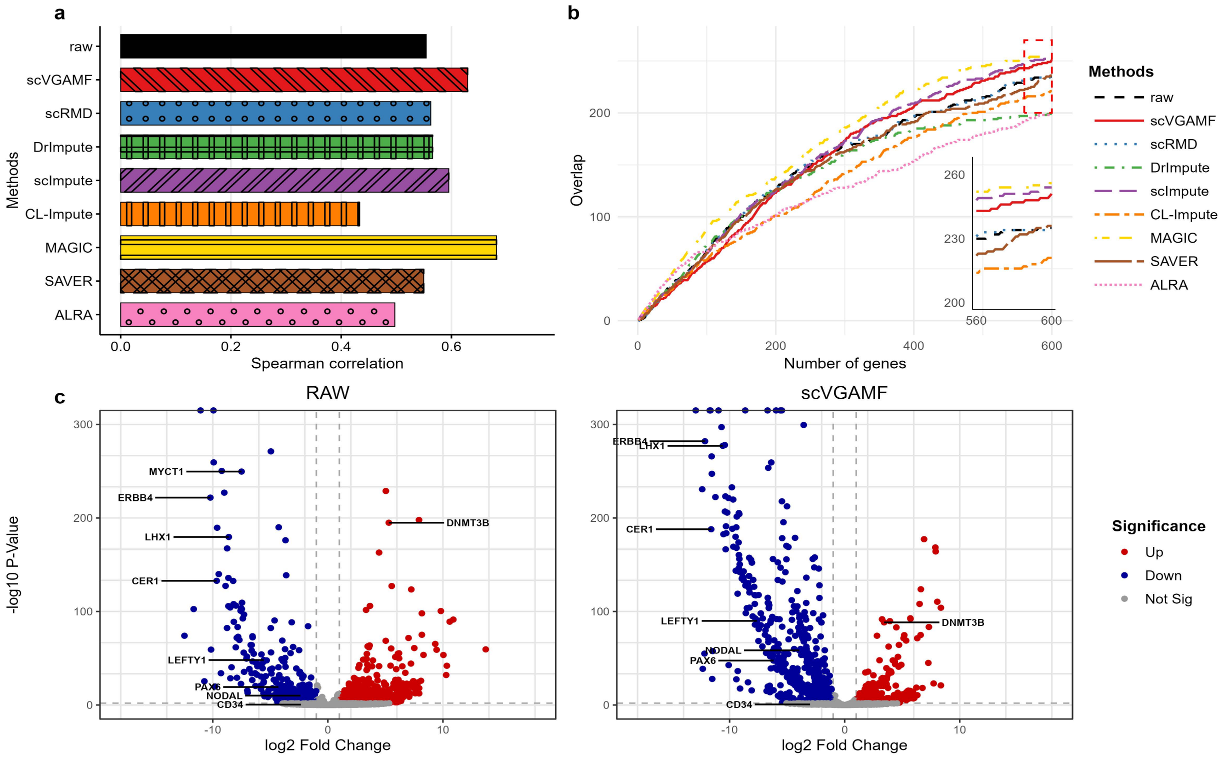

Fig. S5: Differential-expression-based evaluation of imputation accuracy. (a) Spearman correlation of adjusted P-values between bulk and single-cell data (raw or imputed). (b) Overlap between single-cell DE genes and the gold-standard set. (c) Volcano plots comparing DE genes in raw data and scVGAMF-imputed data.

References

[1] Boyd S, Parikh, N, Chu E, et al. Distributed Optimization and Statistical Learning via the Alternating Direction Method of Multipliers. Foundations and Trends in Machine Learning 2011;**3**:1–122. https://doi.org/10.1561/2200000016.

[2] Lange K, Hunter D R, Yang I. Optimization Transfer Using Surrogate Objective Functions. JCGS 2000;**9**:1-20. https://doi.org/10.1080/10618600.2000.10474858.

[3] Li W, Li J. An accurate and robust imputation method scImpute for single-cell RNA-seq data. Nature communications 2018;**9**:997. https://doi.org/10.1038/s41467-018-03405-7.

[4] Huang M, Wang J, Torre E, et al. SAVER: gene expression recovery for single-cell RNA sequencing. Nature methods 2018;**15**:539-542. https://doi.org/10.1038/s41592-018-0033-z.

[5] Zheng W, Min W, Wang S. TsImpute: an accurate two-step imputation method for single-cell RNA-seq data. Bioinformatics 2023;**39**:btad731. https://doi.org/10.1093/bioinformatics/btad731.

[6] Dijk D, Sharma R, Nainys J, et al. Recovering gene interactions from single-cell data using data diffusion. Cell 2018;**174**:716-729. https://doi.org/10.1016/j.cell.2018.05.061.

[7] Gong W, Kwak I Y, Pota P, Koyano-Nakagawa N, Garry D J. DrImpute: imputing dropout events in single cell RNA sequencing data. BMC bioinformatics 2018;**19**:220. https://doi.org/10.1186/s12859-018-2226-y.

[8] Chen C, Wu C, Wu L, Wang X, Deng M, Xi R. scRMD: imputation for single cell RNA-seq data via robust matrix decomposition. Bioinformatics 2020;**36**:3156-3161. https://doi.org/10.1093/bioinformatics/btaa139.

[9] Pan X, Li Z, Qin S, et al. ScLRTC: imputation for single-cell RNA-seq data via low-rank tensor completion. BMC Genomics 2021;**22**:860. https://doi.org/10.1186/s12864-021-08101-3.

[10] Linderman GC, Zhao J, Roulis M, et al. Zero-preserving imputation of single-cell RNA-seq data. Nat Commun 2022;**13**:192. https://doi.org/10.1038/s41467-021-27729-z.

[11] Shi Y, Wan J, Zhang X, et al. CL-Impute: a contrastive learning-based imputation for dropout single-cell RNA-seq data. Comput Biol Med 2023;**164**:107263. https://doi.org/10.1016/j.compbiomed.2023.107263.

[12] Inoue Y. scVGAE: A Novel Approach using ZINB-Based Variational Graph Autoencoder for Single-Cell RNA-Seq Imputation. arXiv preprint arXiv:2403.08959 2024. https://doi.org/10.48550/arXiv.2403.08959.

[13] Wu T, Hu E, Xu S, et al. clusterProfiler 4.0: a universal enrichment tool for interpreting omics data. Innovation (Camb) 2021;**2**:100141. https://doi.org/10.1016/j.xinn.2021.100141.

[14] Yasunaga M, Tada S, Torikai-Nishikawa S, et al. Induction and monitoring of definitive and visceral endoderm differentiation of mouse ES cells. Nat Biotechnol 2005;**23**:1542–1550. https://doi.org/10.1038/nbt1167.

[15] Czyz J, Wobus A. Embryonic stem cell differentiation: the role of extracellular factors. Differentiation 2001;**68**:167–174. https://doi.org/10.1046/j.1432-0436.2001.680404.x.

[16] Wang P, McKnight KD, Wong DJ, et al. A molecular signature for purified definitive endoderm guides differentiation and isolation of endoderm from mouse and human embryonic stem cells. Stem Cells Dev 2012;**21**:2273-2287. doi:10.1089/scd.2011.0416.

[17] J A Hartigan, M A Wong. Algorithm AS 136: A K-Means Clustering Algorithm. Journal of the Royal Statistical Society: Series C (Applied Statistics) 1979;**28**: 100–108. https://doi.org/10.2307/2346830.

[18] Ji Z, Ji H. TSCAN: pseudo-time reconstruction and evaluation in single-cell RNA-seq analysis. Nucleic Acids Research 2016;**44**: e117. https://doi.org/10.1093/nar/gkw430.

[19] Love M I, Huber W, Anders S. Moderated estimation of fold change and dispersion for RNA-seq data with DESeq2. Genome Biology 2014;**15**:550. https://doi.org/10.1186/s13059-014-0550-8.
